# Fine-Grained Assessment of COVID-19 Severity Based on Clinico-Radiological Data Using Machine Learning

**DOI:** 10.3390/ijerph191710665

**Published:** 2022-08-26

**Authors:** Haipeng Liu, Jiangtao Wang, Yayuan Geng, Kunwei Li, Han Wu, Jian Chen, Xiangfei Chai, Shaolin Li, Dingchang Zheng

**Affiliations:** 1 Research Centre for Intelligent Healthcare, Coventry University, Coventry CV1 5FB, UK; 2 Scientific Research Department, HY Medical Technology, B-2 Building, Dongsheng Science Park, Beijing 100192, China; 3 Department of Radiology, The Fifth Affiliated Hospital, Sun Yat-sen University, Zhuhai 519000, China; 4 College of Engineering, Mathematics and Physical Sciences, Streatham Campus, University of Exeter, North Park Road, Exeter EX4 4QF, UK; 5 Guangdong Provincial Key Laboratory of Biomedical Imaging, The Fifth Affiliated Hospital, Sun Yat-sen University, Zhuhai 519000, China

**Keywords:** COVID-19, lesion volume measurement, clinico-radiological features, machine learning, fine-grained classification

## Abstract

Background: The severe and critical cases of COVID-19 had high mortality rates. Clinical features, laboratory data, and radiological features provided important references for the assessment of COVID-19 severity. The machine learning analysis of clinico-radiological features, especially the quantitative computed tomography (CT) image analysis results, may achieve early, accurate, and fine-grained assessment of COVID-19 severity, which is an urgent clinical need. Objective: To evaluate if machine learning algorithms using CT-based clinico-radiological features could achieve the accurate fine-grained assessment of COVID-19 severity. Methods: The clinico-radiological features were collected from 78 COVID-19 patients with different severities. A neural network was developed to automatically measure the lesion volume from CT images. The severity was clinically diagnosed using two-type (severe and non-severe) and fine-grained four-type (mild, regular, severe, critical) classifications, respectively. To investigate the key features of COVID-19 severity, statistical analyses were performed between patients’ clinico-radiological features and severity. Four machine learning algorithms (decision tree, random forest, SVM, and XGBoost) were trained and applied in the assessment of COVID-19 severity using clinico-radiological features. Results: The CT imaging features (CTscore and lesion volume) were significantly related with COVID-19 severity (*p* < 0.05 in statistical analysis for both in two-type and fine-grained four-type classifications). The CT imaging features significantly improved the accuracy of machine learning algorithms in assessing COVID-19 severity in the fine-grained four-type classification. With CT analysis results added, the four-type classification achieved comparable performance to the two-type one. Conclusions: CT-based clinico-radiological features can provide an important reference for the accurate fine-grained assessment of illness severity using machine learning to achieve the early triage of COVID-19 patients.

## 1. Introduction

During the COVID-19 pandemic, it was observed that the mortality was significantly higher in severe and critical cases [1]. After the occurrence of the symptoms of severe acute respiratory infection, some patients rapidly developed acute respiratory distress syndrome (ARDS) and other serious complications, which are followed by multiple organ failure [2]. Therefore, early diagnosis of severe and critical cases could optimize the allocation of medical resources, ensure early intervention for severe and critical patients, and finally reduce the mortality of COVID-19 [3]. 

Clinical features and laboratory data provided important references for assessing the severity of COVID-19 [2,4]. Some physiological parameters such as respiratory rate (RR) and oxygen saturation (SpO2) have been suggested as the indicators of severe and critical cases in clinical guidelines [5]. Some metabolic disturbances and inflammatory dysfunctions [6], such as lymphopenia [7] and a high level of lactate dehydrogenase [8], have also been proven as strong indicators of the severity of COVID-19. Additionally, some clinical comorbidities such as kidney diseases are related with the severity and mortality of COVID-19 [9,10]. The above indicators do provide an informative reference for COVID-19 severity assessment, but they could not directly reflect the severity of the lesion and are insufficient for reliable severity classification due to the complexity of pathophysiology which is patient-specific. 

Computed tomography (CT) imaging has been commonly applied in the diagnosis of COVID-19 [11]. With the deterioration of COVID-19 after the onset of symptoms, specific radiological characteristics such as consolidation, bilateral and peripheral disease, greater total lung involvement, linear opacities, “crazy-paving” pattern and the “reverse halo” sign, can be clearly observed on CT images [12]. In particular, the lesion volume automatically segmented from CT images showed a strong correlation to the prognostic severity of the COVID-19 illness [13]. It has been suggested that clinico-radiological features could indicate the severity of COVID-19 and be applied in the triage of patients [14,15].

Furthermore, based on the clinico-radiological features, various artificial intelligence (AI) methods, especially the machine learning algorithms, have been applied in the assessment of the severity of COVID-19 [16]. Shi et al. developed a least absolute shrinkage and selection operator (LASSO) logistic regression model to predict the prognostic severity of COVID-19 using clinico-radiological data. The authors found that the deep learning LASSO model was more efficient in predicting the severity of COVID-19 than the quantitative CT parameters and the pneumonia severity index (PSI) [15]. Zhang et al. developed a machine learning model using a support vector machine (SVM) to predict the severity of COVID-19 based on 28 clinical features extracted from the results of blood and urine tests [17]. Tang et al. developed a random forest (RF) machine learning model to classify the severity (non-severe vs. severe) of COVID-19 infection based on the quantitative radiological features extracted from CT images, and concluded that the RF-based model can achieve automatic assessment of COVID-19 severity [18]. It was suggested that the lesion volume derived from the automatic quantitative analysis of CT images can be applied in the machine learning model to assess the severity of COVID-19 based on the clinico-radiological features [19]. 

However, the majority of existing studies are focused on the binary classification between severe and non-severe cases. The accurate detection and triage of severe and critical cases plays an important role in reducing the mortality of COVID-19 [3]. Therefore, more fine-grained classification of severity deserves further investigation. Additionally, in existing studies using machine learning algorithms, the clinico-radiological features related with severity have not been comprehensively analyzed from a clinical and pathophysiological perspective. There is a high need to comprehensively evaluate the clinico-radiological features related to the severity of COVID-19 and develop the machine learning methods to achieve the early fine-grained assessment of COVID-19 severity. 

In this study, based on the analysis of CT images using neural network, we aim to find the clinico-radiological features significantly related with the severity of COVID-19, and evaluate if machine learning algorithms could achieve the accurate fine-grained assessment of COVID-19 severity using clinico-radiological features. This work will pave the way for the early triage of COVID-19 patients, which will optimize the allocation of medical resources during the pandemic and effectively decrease the mortality of COVID-19, especially among severe and critical cases. 

## 2. Materials and Methods

### 2.1. Patient Recruitment

A total of 78 patients (44 females, 34 males, mean ± standard deviation [SD] of age: 50.3 ± 14.5 years) with a confirmed SARS-CoV-2 laboratory test between 18 January 2020, and 5 March 2020, in Zhuhai, China were recruited in a retrospective, single-center study. A patient was confirmed as positive by high-throughput sequencing or real-time reverse-transcriptase polymerase-chain-reaction (RT-PCR) assay of nasal and pharyngeal swab specimens. The RT-PCR test kits were manufactured by Shanghai Zhijiang Biotechnology Co. (Shanghai, China). This study was approved by the ethics committee of the Fifth Affiliated hospital of Sun Yat-Sen University, and the requirement for informed consent was waived. The private information of patients was anonymized by the investigators after data collection. The inclusion criteria were: (a) patients with positive novel coronavirus nucleic acid antibody test and confirmed by the Centers for Disease Control (CDC); (b) age >= 14. Patients with comorbidities of other acute respiratory diseases were excluded. 

### 2.2. Clinical Data Collection

Clinical data was collected by chart review. The patients were classified into four types according to patients’ most severe conditions, using the Diagnosis and Treatment Plan of COVID-19 issued by National Health Commission (7th ed.): (1) mild type: minimal clinical symptoms without pneumonia in imaging; (2) regular type: fever, respiratory and other symptoms with pneumonia in imaging; (3) severe type: respiratory distress, respiratory rate ≥30 times/min; in resting state, oxygen saturation ≤93%; PaO2/FiO2 ≤ 300 mmHg; (4) critical type: respiratory failure requiring mechanical ventilation, shock and other organ failure requiring ICU monitoring and treatment [20].

### 2.3. CT Imaging Protocol

All scans were performed with patients in the supine position during end-inspiration without intravenous contrast on three CT scanners: uCT 760, uMI 780 scanners (United Imaging; Shanghai, China) and Precision 32 (CAMPO Imaging; Shenyang, China). Images were obtained from the apex to lung bases, using a standard dose protocol, reconstructed at 1.0 mm/1.1 mm slice thickness, with 0.7 mm increment, 512 × 512 mm and a sharp reconstruction kernel. The lung window width and level settings were 1500 Hounsfield units (HU) and −600 HU. 

### 2.4. CT Image Analysis

#### 2.4.1. CTscore from Visual Quantitative Evaluation

The CTscore was retrospectively obtained by visual quantitative evaluation of acute lung inflammatory lesion on CT images. Two radiologists blinded to the clinical information reviewed all images independently. In each lobe, the score was calculated from the percentage of total lesion areas as 0 (0%), 1 (1–25%), 2 (26–50%), 3 (51–75%), or 4 (76–100%). For each subject, by adding the scores of five lung lobes, the total severity score (CTscore) ranged from 0 to 20. The final score of each case was decided by a third experienced thoracic radiologist. The details can be found in our published work [21].

#### 2.4.2. MT-HRNet-3d Neural Network for Lesion Volume Measurement

**Figure 1:**
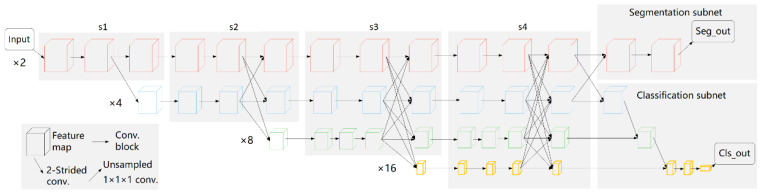
The network architecture of MT-HRNet-3d, composed of a simple stem, a main body, as well as the segmentation and classification subnets. The main body consists of four stages of parallel high-to-low resolution subnetworks with repeated information exchange across multi-resolution subnetworks (multi-scale fusion). ×n denotes the down-sampling ratio of the resolution representation to the input image, and the numbers of blocks of each stage module are [2,2,3,3] in [×2, ×4, ×8, ×16] resolution levels, respectively. In the segmentation subnet, only the highest resolution representations are concatenated for the medical lesion segmentation, while in the classification subnet, all the high-to-low resolution representations are aggregated for multi-class classification. Seg_out and Cls_out are the outputs of two subnets. In this study, the lesion volume reconstructed from Seg_out result was used as an input of machine learning algorithm of severity classification.

The lesion volume was calculated based on an AI algorithm which was developed by HY Medical Technology Co., Ltd (Beijing, China). The main neural network framework is detailed in Figure 1.

The original high-resolution network, named HRNetV1 [22], maintains high-resolution representations by exchanging information across multi-resolution subnetworks. HRNetV2 [23] explores the representations from all the high-to-low resolution parallel convolutions other than only the high-resolution representations in HRNetV1, which adds a small overhead and leads to stronger high-resolution representations. To achieve the three-dimensional (3d) medical image classification and segmentation, we modified the existing networks and built a 3d high-resolution network, named MT-HRNet-3d (3d Multi-Task High-Resolution Network). 

As shown in Figure 1, the MT-HRNet-3d network is composed of a simple stem, a main body, as well as the segmentation and classification subnets. The simple stem consists of one 2-strided convolutions decreasing the resolution, remaining the scale of the highest resolution at 2. The main body consists of four stages of parallel high-to-low resolution subnetworks, outputting the high-to-low resolution feature maps through repeatedly fusing the representations produced by the high-to-low subnetworks. With segmentation and classification subnets, the network is targeted for the 3d medical image classification and segmentation. In this study, we used the 3d lesion volume reconstructed from segmented masks of lesions (i.e., the output of segmentation subnet) as an input for the machine learning algorithm of severity classification. The segmentation subset had been trained and validated based on the consensus manual segmentation results of three radiological experts as the gold standard.

#### 2.4.3. Modification of Neural Network

Following [22], the widths (number of channels) of the convolutions of the four resolutions were C, 2C, 4C, and 8C, where C was set to 32. In order to improve the computational efficiency, a simple modification in the main body was made by including each branch in the multi-resolution group convolution with different residual blocks to save the memory. Specifically, the four high-to-low resolution branches contain 2, 2, 3, and 3 blocks, respectively, where the 2nd, 3rd, and 4th stages contain 1, 2, and 2 multi-resolution modules. 

Notably, we considered the HRNet as a naturally multi-task learning framework for the segmentation and classification and believe that the good feature representations in different resolution can be learned by related multi-task learning. Therefore, the segmentation and classification subnets were added simultaneously. Instead of all the high-to-low resolution representations, only the highest resolution representations were concatenated for the medical lesion segmentation, resulting in fine segmentation contours. In the classification subnet, all the high-to-low resolution representations were aggregated for multi-class classification.

#### 2.4.4. Multi-Task Loss Function

A MT-HRNet-3d network has two sibling output subnets. The classification subnet outputs a discrete probability distribution (per Image), *p* = (p0, ..., pK), over K + 1 categories. As usual, *p* is computed by a softmax over the K + 1 outputs of a linear classifier. The segmentation subnet outputs a probability segmentation map with the same resolution as the input feature map of the main body, then the segmentation map is upsampled (2 times) to the input size by trilinear upsampling. To jointly train the model, the related multi-task loss is defined as,

*L* = *L*_*CE*_ + *λ1_mask_L_Dice_* (1)

in which *L_CE_* is the cross-entropy loss for the image-level classification. The second task loss, *L_Dice_*, is the dice loss, defined over the probability segmentation map and the pixel-level labeled mask. The indicator function *1_mask_* evaluates to 1 when the lesion mask is labeled and 0 otherwise. The hyper-parameter λ in Equation (1) controls the balance between the two task losses, usually set to 1 based on our experience. By sharing the information during training, the multi-task learning approach can improve data efficiency, reduce overfitting, and therefore enhance the overall efficiency of the algorithm in achieving an accurate estimation of lesion area.

### 2.5. Data Cleansing

The full clinico-radiological features are listed in Table A1. Before data cleansing, the columns unrelated with patients’ pathophysiological information (e.g., ‘MedNum’, ‘No’) and columns that have no data (e.g., ‘LVEF’, ‘SO2’, ‘PO2’, ‘YHZS’) were excluded. Then, the features with missing recordings in more than 10 subjects (e.g., ‘Onset2severity’) were removed. Finally, 59 clinico-radiological features were used for analysis. 

### 2.6. Statistical Analysis

To evaluate if the clinico-radiological features, especially the CT imaging features (CTscore and VRmax), were significantly related with the prognostic severity of COVID-19, statistical analysis was performed between the clinico-radiological features and the severity in the two-type and four-type classifications, respectively. 

For quantitative features, firstly, the Shapiro–Wilk test was performed to examine if the data follow normal distribution in each severity subgroup. If normal distribution was satisfied (i.e., *p* > 0.05 in Shapiro–Wilk test), the *t*-test was performed in the two-type classification to examine if there was a significant difference in the feature between the severe and non-severe patients. For the four-type classification, Levene’s test was performed to examine the homogeneity of variance among subgroups. For any feature where the homogeneity of variance was satisfied or violated (i.e., *p* > 0.05 or *p* *≤* 0.05 in Levene’s test), the analysis of variance (ANOVA) and Welch’s ANOVA was performed, respectively to examine if there was a significant different in the feature among patients with different severities.

For ordinal features and the quantitative features that did not follow normal distribution (i.e., *p* *≤* 0.05 in Shapiro–Wilk test), the non-parametric tests were performed. The Mann–Whitney U test and Kruskal–Wallis H test were performed as the alternatives to *t*-test and ANOVA in the two-type and four-type classifications, respectively.

For categorical features, the differences between rates were tested by Chi-squared (χ^2^) or Fisher’s exact tests, if appropriate. A *p*-value less than 0.05 was considered as statistically significant in all the comparisons.

### 2.7. Machine Learning Algorithms

The data were processed using four different classification algorithms: decision tree, random forest, SVM, and XGBoost. As aforementioned, 59 clinico-radiological features (bold in Table A1) were selected. We applied feature engineering which is commonly used in machine learning to derive the feature set for the training of machine learning algorithms [24]. First, constant and quasi-constant features (i.e., with the same value or limited variations among all patients) were removed. Second, Pearson’s correlation coefficient was calculated between different features. For correlated features, only the most indicative one was kept, with others removed to reduce information redundancy. Third, statistical methods that calculate mutual information is employed to further remove features with redundant information. The algorithm was automatic. Finally, 37 features were input for algorithmic training. 

The estimation of the patient’s severity was the output, based on the two-type (non-severe and severe) and four-type classifications (mild, regular, severe, critical), respectively. Firstly, the testing dataset was separated randomly as 10% of the original dataset. The remaining data was split into the training set (80%) and testing set (20%) for the 5-fold cross validation. To investigate the significance of CT analysis results in assessing the severity, the assessment was performed with VRmax and CTscore included and excluded, respectively. The precision, recall, and F1 values were used for the quantitative comparison of results. The results of two-type and four-type classifications were also compared to initially examine the performance of fine-grained classification of COVID-19 severity. 

To illustrate the contribution of different features in the decision made by machine learning methods, we used the local interpretable model-agnostic explanations (LIME) which is a common method for explaining black-box models, i.e., models whose inner logic is hidden and not clearly understandable [25]. On the standardized *p*-dimensional dataset where *p* is the number of retained features, LIME performs ridge regression, which is trained in a weighted fashion, i.e., each point contributes to the model according to its weight. In the resultant model, the coefficient of a feature reflects its contribution in the classification: the higher the coefficient, the bigger the variation in the output when the feature is changed. The sign of the coefficient shows the direction of the variation in the output [25]. 

## 3. Results

### 3.1. Statistical Analysis

#### 3.1.1. Significant Clinico-Radiological Features in Two-Type Classification

The data distribution is balanced in two-type (57 non-severe, 21 severe) classification but there is a lack of mild cases in four-type classifications (57 regular, 16 severe, 5 critical). In the quantitative variables, the normal distribution was satisfied in the following features: BMI, cTnlTimes, LYM1, ALB1, and ALB2 (*p* > 0.05 for all in Shapiro–Wilk test). The clinico-radiological features that are significantly different in severe and non-severe cases are shown in Table 1.

#### 3.1.2. Significant Clinico-Radiological Features in Four-Type Classification

In the quantitative variables, the normal distribution was satisfied in the following features: weight, BMI, cTnlTimes, LYM1, ALB1, and ALB2 (*p* > 0.05 for all in Shapiro–Wilk test), where the homogeneity of variance was satisfied in all (*p* > 0.05 in Levene’s test) except cTnlTimes (*p* < 0.001). The clinico-radiological features that are significantly different among the patients with different severities are shown in Table 2. In both the two-type and four-type classifications, the CT imaging features were significantly related with the severity of COVID-19.

### 3.2. Machine Learning

#### 3.2.1. Role of CT Images Analysis in Two-Type Classification

After feature engineering, we found out that NtproBNP, LYM, LDH, and CRP were the four most indicative ones of the 37 remaining features. As shown in Table 3, overall, the addition of AI-assisted CT image analysis results (radiological features) did not improve the accuracy of the algorithms in two-type classification. Only Decision algorithms showed mild improvement in F1 value with worsening in other metrics, while XGBoost and Random Forest showed some worsening. The SVM results were unaffected. 

#### 3.2.2. Role of CT Images Analysis in Four-Type Classification

In Table 3, it can be observed that with the addition of AI-assisted CT images analysis, the accuracy of the estimation has been improved in Decision Tree, Random Forest, and XGBoost but not in SVM.

#### 3.2.3. Patient-Specific Analysis of Significant Clinico-Radiological Features in Machine Learning

**Figure 2:**
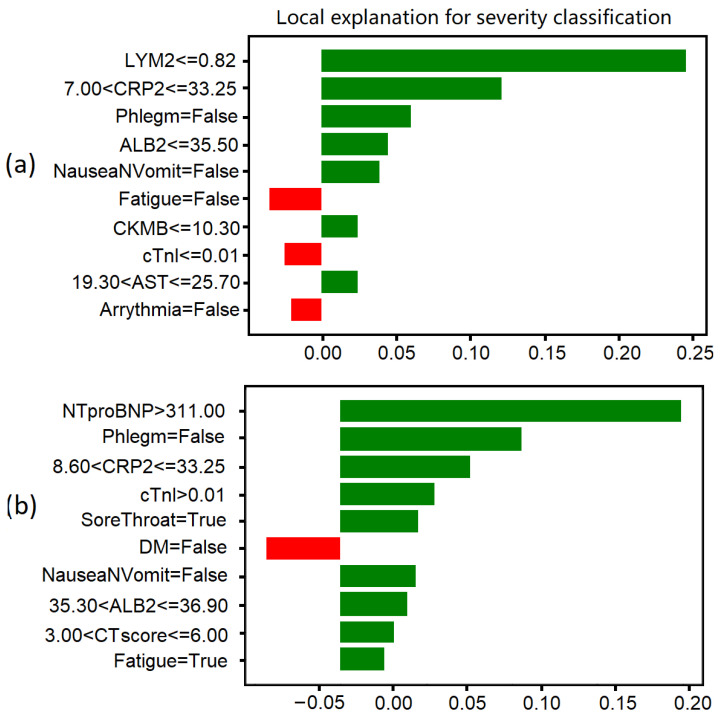
Comparison of clinico-radiological features in different cases based on local interpretable model-agnostic explanations (LIME). (**a**) A correctly classified severe cases in two-type classification. (**b**) A correctly classified severe case in four-type classification. The values on x axis are the coefficients of the ridge regression model fitted locally to the predictions from the original model. Features in green have a positive contribution to the prediction (increasing the probability of turning severe), and features in red have a negative effect on the prediction (decreasing the probability of turning severe).

Figure 2a shows a correctly classified severe case in two-type classification. The existence of arrhythmia and fatigue, which may be related with cardiac diseases and the development of COVID-19, indicate higher severity. The phlegm showed the opposite relationship with severity. A possible explanation is that the lack of phlegm will make it difficult for the patient to expel the sputum from the lung, which will deteriorate the affection in the lung and lead to higher severity. Figure 2b shows a correctly classified severe case in four-type classification. It can be seen that the key clinico-radiological features are different from the two-type classification in Figure 2a, where the CTscore becomes a key factor.

**Figure 3:**
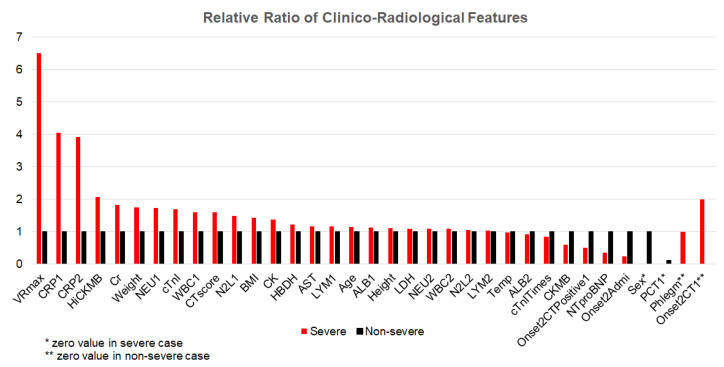
Comparison of clinico-radiological features between a non-severe case and a severe case. The values of features in the severe case are in relative value of corresponding values in the non-severe case, except those with zero values (Sex, PCT1, Phlegm, and Onset2CT1).

Figure 3 shows two cases with similarity in many clinical features. With the machine learning method, they have been accurately classified as non-severe and severe, and regular and severe, in the two-type and four-type classifications, respectively. It can be observed that the VRmax and CTscore showed significant difference between the two cases, which improved the accuracy of classification. In particular, in these two cases, some clinical features did not show strong efficiency (e.g., NTproBNP is even lower in the severe case) but the VRmax correctly indicates the right trend (i.e., higher in the severe case) with the highest relative ratio between the severe and non-severe cases, showing high robustness in assessing COVID-19 severity against patient-specific variability in pathophysiological features.

## 4. Discussion

### 4.1. Comparison between Statistical Analysis and Machine Learning Results

In statistical analysis, the CT image analysis results (CTscore and VRmax) were significant features of the severity in both two-type (*p* < 0.001 for both) and four-type (*p* < 0.001 for both) classifications. The inclusion of CT image analysis results significantly improved the accuracy of machine learning algorithms in evaluating the severity of COVID-19 using four-type classification. These results commonly suggest that CT image analysis could provide important reference for the fine-grained assessment of illness severity to achieve the early triage of COVID-19 patients. Compared with the statistical analysis, the machine learning algorithms provided the patient-specific quantitative evaluation of different clinico-radiological features regarding the illness severity, providing a more detailed reference for the accurate diagnosis and treatment of COVID-19.

### 4.2. Quantitative CT Image Analysis: A Key to COVID-19 Severity Assessment

It has been suggested that CT image analysis could improve the machine learning algorithm to achieve a higher accuracy in diagnosing COVID-19 than the clinical COVID-19 reporting and data system [26]. In particular, the lesion volume segmented and quantified using deep learning showed a strong potential in the prediction of COVID-19 severity [27]. Furthermore, the ratio of compromised lung volume (sum of poorly and non-aerated volumes) has been observed to be an accurate outcome predictor of the risk of oxygenation support and intubation, and in-hospital mortality (*p* < 0.001 for all in logistic regression) [28]. Therefore, the quantitative CT image analysis (especially the lesion volume) plays a key role in the assessment of the severity of COVID-19 and provides important reference to guide the clinical triage and intervention. 

According to the model interpretation (two-type XGBoost classification), the model unveils several important clinic-radiological features such as NTproBNP. As a result, among all those important features, the CTscore improves the performance only moderately. For the four-type classification, the CTscore improves in all classification algorithms but not in SVM due to its structure. The SVM model decides its hyperplane based on all the features. The CTscore does have influence on the decision of the hyperplane, but its influence is not strong enough to demonstrate its efficacy on the final decision. 

### 4.3. Fine-Grained Severity Assessment of COVID-19 Using Machine Learning: Clinical Significance

Considering the current epidemic of COVID-19 and the high risk of its recurrence, the early detection of severe COVID-19 illness is an urgent clinical need. Currently, both the two-type [29] and four-type [30] classification standards are widely used in the triage of COVID-19 patients. In the latest guidelines of Chinese Medical Association, the severe and critical cases of COVID-19 were unified as “severe” due to the rapid exacerbation of severe cases, the highly diverse “time window”, and the difficulty in detecting critical cases which may lead to the delayed treatment [31]. The time lengths from illness onset to severe symptoms such as dyspnea (95% confidence interval (CI): 4.0–9.0 days) or critical symptoms such as sepsis (95% CI: 7.0–13.0 days) are patient-specific and covers a wide range [1]. Additionally, the RT-PCT test has low sensitivity in the first 3–5 days of affection [32]. Therefore, the early and accurate detection of clinical risks based on the four-type classification could play an important role in guiding the clinical intervention for the severe and critical cases towards more appropriate management. Additionally, due to the rapidly growing imbalance between supply and demand for medical resources, the fair allocation of medical resources has become an urgent clinical need in many countries [33]. In Table 3, with CT analysis results added, the four-type classification achieved comparable or even better performance in some metrics than the two-type classification, especially in Random Forest and XGBoost algorithms, which indicates that the fine-grained classification of COVID-19 severity could achieve comparable performance to the widely used binary classification on the same dataset while providing a more detailed reference for clinical diagnosis, treatment, and management. With accurate estimation of severity, this machine learning method could be used to guide the allocation of limited medical resources.

### 4.4. Limitations and Future Directions

Firstly, only 78 patients were included in this single-center, retrospective pilot study. Secondly, the distributions of age and severity were imbalanced in these cases. There were only five cases aged 19–30 years and only one case aged less than 18 years. The majority (57.5%) of the included cases were moderate. Only five critical cases were included. There was a lack of mild cases. During the early outbreak period, the patients with mild symptoms often had delayed hospital admission due to the lack of awareness. A balanced training dataset is important to improve the reliability of the algorithm and its applicability in different cohorts. Additionally, it has been found the severity of COVID-19 was related with other physiological factors including pregnancy [14], and the comorbidity of chronical respiratory diseases, cardiovascular diseases [10], diabetes [34], and cancer [35]. Therefore, the results derived by the machine learning models need to be validated in large-scale datasets with more balanced data distribution. In future studies, more clinical datasets could be included to cover a wider range of age and severity, as well as those in different physiological and pathological conditions.

## 5. Conclusions

The results of quantitative CT image analysis were significantly related to the severity of COVID-19. The clinico-radiological features including the CT image analysis results can provide an important reference for the fine-grained assessment of illness severity using machine learning to achieve the early triage of COVID-19 patients.

## Figures and Tables

**Table 1:** Statistically significant (*p* value < 0.05) clinico-radiological features of severity in two-type classification.

Clinico-Radiological Features	*p*-Value	Statistical Test
cTnITimes	<0.001	*t*-test
LYM1	0.001	*t*-test
ALB1	0.003	*t*-test
ALB2	<0.001	*t*-test
Age	0.006	Mann–Whitney U test
CTscore	<0.001	Mann–Whitney U test
VRmax	<0.001	Mann–Whitney U test
cTnI	<0.001	Mann–Whitney U test
AST	0.023	Mann–Whitney U test
LDH	<0.001	Mann–Whitney U test
HBDH	<0.001	Mann–Whitney U test
NTproBNP	<0.001	Mann–Whitney U test
N2L1	0.006	Mann–Whitney U test
CRP1	<0.001	Mann–Whitney U test
LYM2	<0.001	Mann–Whitney U test
N2L2	<0.001	Mann–Whitney U test
CRP2	<0.001	Mann–Whitney U test
cTnlCKMBOrdinal1	0.001	Chi-squared test with Fisher’s exact test
CTnlCKMBOrdinal2	0.002	Chi-squared test with Fisher’s exact test
Phlegm	0.002	Chi-squared test with Fisher’s exact test
Fatigue	0.004	Chi-squared test with Fisher’s exact test
DM	0.042	Chi-squared test with Fisher’s exact test

**Table 2:** Statistically significant (*p* value < 0.05) clinico-radiological features of severity in four-type classification.

Clinico-Radiological Features	*p*-Value	Statistical Test
cTnITimes	0.005	Welch’s ANOVA
LYM1	0.001	ANOVA
ALB1	0.003	ANOVA
ALB2	<0.001	ANOVA
Age	0.003	Kruskal–Wallis H test
CTscore	<0.001	Kruskal–Wallis H test
VRmax	<0.001	Kruskal–Wallis H test
cTnI	<0.001	Kruskal–Wallis H test
LDH	0.001	Kruskal–Wallis H test
HBDH	0.001	Kruskal–Wallis H test
NTproBNP	<0.001	Kruskal–Wallis H test
N2L1	0.004	Kruskal–Wallis H test
CRP1	<0.001	Kruskal–Wallis H test
LYM2	<0.001	Kruskal–Wallis H test
N2L2	<0.001	Kruskal–Wallis H test
CRP2	<0.001	Kruskal–Wallis H test
cTnlCKMBOrdinal1	<0.001	Chi-squared test with Fisher’s exact test
CTnlCKMBOrdinal2	<0.001	Chi-squared test with Fisher’s exact test
Phlegm	0.010	Chi-squared test with Fisher’s exact test
Fatigue	<0.001	Chi-squared test with Fisher’s exact test
DM	0.01	Chi-squared test with Fisher’s exact test
Smoking	0.019	Chi-squared test with Fisher’s exact test
Lung	0.015	Chi-squared test with Fisher’s exact test

**Table 3:** Estimation results of machine learning algorithms in two-type classification *.

	Classifier	Without CT Image Analysis				With CT Image Analysis			
		Cross Validation	Test Set			Cross Validation	Test Set		
		F1	Precision	Recall	F1	F1	Precision	Recall	F1
two-type classification	Decision Tree	0.57	0.89	0.75	0.86	**0.61**	0.67	0.50	0.57
	Random Forest	0.67	0.67	0.25	0.40	0.64	0.67	0.25	0.40
	SVM	0.67	0.67	0.25	0.40	0.67	0.67	0.25	0.40
	XGBoost	0.71	0.78	1.00	0.80	0.68	0.78	1.00	0.80
four-type (fine-grained) classification	Decision Tree	0.53	0.13	0.25	0.17	**0.77**	**0.56**	**0.50**	**0.52**
	Random Forest	0.55	0.50	0.42	0.36	**0.78**	**0.76**	**0.75**	**0.75**
	SVM	0.52	0.50	0.42	0.36	0.52	0.50	0.42	0.36
	XGBoost	0.49	0.50	0.42	0.36	**0.80**	**0.86**	**0.75**	**0.71**

* The bold font shows the improvements in performance due to CT image analysis.

## Data Availability

Not applicable.

## Appendix A

**Table A1:** Clinico-radiological features.

Feature	Note	Category
MedNum	Number of Medical Insurance	x
No	Number	x
**Sex**	Sex	Basic information
**Age**	Age	Basic information
**AgeG1**	Age > 50	Basic information
**Height**	Height	Basic information
**Weight**	Weight	Basic information
**BMI**	Body Mass Index	Basic information
**Temp**	Body temperature	
**Severity03**	Severity 0–3: mild, regular, severe, critical	Categories of severity
**Severity01**	Severity 0–1: non-severe, severe	Categories of severity
**CTscore**	Peak CT scores	Visual quantification on CT images
**VRmax**	The highest value of the affected Volume Ratio among all CT scans for the particular patient.	Detection results derived by algorithm
**cTnITimes**	Time of cTnI measurement	Reagent Test
**cTnI**	Maximal Cardiac Troponin I (cTnI)	Reagent Test
**cTnlCKMBOrdinal1**	Whether troponin I is over 0.0229 µg/mL or CKMB is over twice of the normal threshold (50 U/L) on admission	Reagent Test
**cTnlCKMBOrdinal2**	Whether troponin I is over 0.0229 µg/mL or CKMB is over twice of the normal threshold (50 U/L) on admission during the hospitalization according to the maximal values	Reagent Test
**AST**	Maximal aminotransferase. AST level in serum is a clinical indicator of myocardial infarction and myocarditis, which is low in normal cases.	Biochemical marker
**LDH**	Lactate dehydrogenase. Increased activity of LDH activity is mainly found in myocardial infarction, acute or chronic hepatitis, and liver cancer.	Biochemical marker
**CK**	Creatine kinase, an indicator of myocardial diseases	Biochemical marker
**CKMB**	Creatine kinase myocardial band, a specific serum marker of cardiomyocyte damage	Biochemical marker
**HBDH**	α-hydroxybutyrate dehydrogenase, which actually reflect the activity of LDH1 and LDH2, which is has diagnostic significance of myocardial and liver diseases	Biochemical marker
**HiCKMB**	Highest level of CKMB	Biochemical marker
**NTproBNP**	N-terminal pro b-type natriuretic peptide, for clinical diagnosis of heart failure	Biochemical marker
**Cr**	Serum creatinine	Biochemical marker
LVEF	left ventricular ejection fraction (maximum) not included in the majority	Color Doppler ultrasonic
**PCT1**	procalcitonin, which increases during bacterial, fungal, and parasitic infections as well as sepsis and multiple organ failure, maximal value on admission	Inflammation marker
**WBC1**	white blood cells on admission	Blood routine examination
**NEU1**	Neutrophils on admission	Blood routine examination
**LYM1**	Lymphocytes on admission	Blood routine examination
**N2L1**	Ratio of granulocytes to lymphocytes on admission	Blood routine examination
**CRP1**	C-reactive protein (CRP) on admission, which rises sharply in plasma due to infection or tissue damage	Inflammation marker
**ALB1**	Albumin (on admission)	Biochemical marker
**PCT2**	Procalcitonin (maximum values for non-severe patients or pre-severe phase of severe patients)	Inflammation marker
**WBC2**	white blood cells (maximum values for non-severe patients or pre-severe phase of severe patients)	Blood routine examination
**NEU2**	Neutrophils (maximum values for non-severe patients or pre-severe phase of severe patients)	Blood routine examination
**LYM2**	Lymphocytes (maximum values for non-severe patients or pre-severe phase of severe patients)	Blood routine examination
**N2L2**	Ratio of granulocytes to lymphocytes (maximum values for non-severe patients or pre-severe phase of severe patients)	Blood routine examination
**CRP2**	C-reactive protein (maximum values for non-severe patients or pre-severe phase of severe patients)	Inflammation marker
**ALB2**	Albumin (maximum values for non-severe patients or pre-severe phase of severe patients)	Biochemical marker
SO2	x	x
PO2	x	x
YHZS	x	x
**Symptom**	Symptoms on admission	Basic symptom
**Fever**		Basic symptom
**Cough**		Basic symptom
**Phlegm**		Basic symptom
**Hemoptysis**		Basic symptom
**SoreThroat**	Sore throat	Basic symptom
**Catarrh**		Basic symptom
**Headache**		Basic symptom
**ChestPain**	Chest pain	Basic symptom
**Fatigue**		Basic symptom
**SoreMuscle**	Sore muscle	Basic symptom
**Stomachache**		Basic symptom
**Diarrhea**		Basic symptom
**PoorAppetie**	Poor appetite	Basic symptom
**NauseaNVomit**	Nausea and Vomit	Basic symptom
**Hypertention**		Medical history
**Hyperlipedia**		Medical history
**DM**	Diabetes mellitus	Medical history
**Lung**	Lung diseases	Medical history
**CAD**	Coronary artery disease	Medical history
**Arrythmia**	Arrythmia	Medical history
**Cancer**	Cancer	Medical history
Onset2Admi	The time period from onset of symptoms to admission	Time
Onset2CT1	The time period from onset of symptoms to the first CT imaging	Time
Onset2CTPositive1	The time period from onset of symptoms to the first CT Positive results	Time
Onset2severity	The time period from onset of symptoms to the first diagnosis of two-type severity	Time
Onset2CTPeak	The time period from onset of symptoms to CT Peak results	Time

Selected clinic-radiological features are in bold. In the paired features (e.g., PCT1 and PCT2), the numbers 1 and 2 indicate the admission value and maximal value during hospitalization, respectively.

## References

[1] Zhou F., Yu T., Du R., Fan G., Liu Y., Liu Z., Xiang J., Wang Y., Song B., Gu X. (2020). Clinical course and risk factors for mortality of adult inpatients with COVID-19 in Wuhan, China: A retrospective cohort study. Lancet.

[2] Gao Y., Li T., Han M., Li X., Wu D., Xu Y., Zhu Y., Liu Y., Wang X., Wang L. (2020). Diagnostic utility of clinical laboratory data determinations for patients with the severe COVID-19. J. Med. Virol..

[3] Sun Q., Qiu H., Huang M., Yang Y. (2020). Lower mortality of COVID-19 by early recognition and intervention: Experience from Jiangsu Province. Ann. Intensive Care.

[4] Wang Y., Wang Y., Chen Y., Qin Q. (2020). Unique epidemiological and clinical features of the emerging 2019 novel coronavirus pneumonia (COVID-19) implicate special control measures. J. Med. Virol..

[5] World Health Organization (2020). Clinical Management of Severe Acute Respiratory Infection (SARI) When COVID-19 Disease Is Suspected: Interim Guidance, 13 March 2020.

[6] Nie S., Zhao X., Zhao K., Zhang Z., Zhang Z., Zhang Z. (2020). Metabolic disturbances and inflammatory dysfunction predict severity of coronavirus disease 2019 (COVID-19): A retrospective study. MedRxiv.

[7] Tan L., Wang Q., Zhang D., Ding J., Huang Q., Tang Y.-Q., Wang Q., Miao H. (2020). Lymphopenia predicts disease severity of COVID-19: A descriptive and predictive study. Signal Transduct. Target. Ther..

[8] Han Y., Zhang H., Mu S., Wei W., Jin C., Tong C., Song Z., Zha Y., Xue Y., Gu G. (2020). Lactate dehydrogenase, an independent risk factor of severe COVID-19 patients: A retrospective and observational study. Aging.

[9] Cheng Y., Luo R., Wang K., Zhang M., Wang Z., Dong L., Li J., Yao Y., Ge S., Xu G. (2020). Kidney disease is associated with in-hospital death of patients with COVID-19. Kidney Int..

[10] Wang X., Fang X., Cai Z., Wu X., Gao X., Min J., Wang F. (2020). Comorbid Chronic Diseases and Acute Organ Injuries Are Strongly Correlated with Disease Severity and Mortality among COVID-19 Patients: A Systemic Review and Meta-Analysis. Research.

[11] Li Y., Xia L. (2020). Coronavirus Disease 2019 (COVID-19): Role of Chest CT in Diagnosis and Management. Am. J. Roentgenol..

[12] Bernheim A., Mei X., Huang M., Yang Y., Fayad Z.A., Zhang N., Diao K., Lin B., Zhu X., Li K. (2020). Chest CT Findings in Coronavirus Disease-19 (COVID-19): Relationship to Duration of Infection. Radiology.

[13] Liu F., Zhang Q., Huang C., Shi C., Wang L., Shi N., Fang C., Shan F., Mei X., Shi J. (2020). CT quantification of pneumonia lesions in early days predicts progression to severe illness in a cohort of COVID-19 patients. Theranostics.

[14] Liu F., Liu H., Hou L., Li J., Zheng H., Chi R., Lan W., Wang D. (2020). Clinico-Radiological Features and Outcomes in Pregnant Women with COVID-19 Pneumonia Compared with Age-Matched Non-Pregnant Women. Infect. Drug Resist..

[15] Shi W., Peng X., Liu T., Cheng Z., Lu H., Yang S., Zhang J., Wang M., Gao Y., Shi Y. (2021). A deep learning-based quantitative computed tomography model for predicting the severity of COVID-19: A retrospective study of 196 patients. Ann. Transl. Med..

[16] Shi F., Wang J., Shi J., Wu Z., Wang Q., Tang Z., He K., Shi Y., Shen D. (2020). Review of Artificial Intelligence Techniques in Imaging Data Acquisition, Segmentation and Diagnosis for COVID-19. IEEE Rev. Biomed. Eng..

[17] Zhang N., Zhang R., Yao H., Xu H., Duan M., Xie T., Pan J., Huang J., Zhang Y., Xu X. (2020). Severity Detection For the Coronavirus Disease 2019 (COVID-19) Patients Using a Machine Learning Model Based on the Blood and Urine Tests. Front. Cell Dev. Biol..

[18] Tang Z., Zhao W., Xie X., Zhong Z., Shi F., Liu J., Shen D. (2020). Severity assessment of coronavirus disease 2019 (COVID-19) using quantitative features from chest CT images. arXiv.

[19] Cai W., Liu T., Xue X., Luo G., Wang X., Shen Y., Fang Q., Sheng J., Chen F., Liang T. (2020). CT Quantification and Machine-learning Models for Assessment of Disease Severity and Prognosis of COVID-19 Patients. Acad. Radiol..

[20] National Health Commission, National Administration of Traditional Chinese Medicine (2020). Translation: Diagnosis and Treatment Protocol for Novel Coronavirus Pneumonia (Trial Version 7). Infect. Microbes Dis..

[21] Li K., Fang Y., Li W., Pan C., Qin P., Zhong Y., Liu X., Huang M., Liao Y., Li S. (2020). CT image visual quantitative evaluation and clinical classification of coronavirus disease (COVID-19). Eur. Radiol..

[22] Sun K., Xiao B., Liu D., Wang J. Deep High-Resolution Representation Learning for Human Pose Estimation. Proceedings of the 2019 IEEE/CVF Conference on Computer Vision and Pattern Recognition (CVPR).

[23] Sun K., Zhao Y., Jiang B., Cheng T., Xiao B., Liu D., Mu Y., Wang X., Liu W., Wang J. (2019). High-resolution representations for labeling pixels and regions. arXiv.

[24] Kuhn M., Johnson K. (2019). Feature Engineering and Selection: A Practical Approach for Predictive Models.

[25] Visani G., Bagli E., Chesani F., Poluzzi A., Capuzzo D. (2022). Statistical stability indices for LIME: Obtaining reliable explanations for machine learning models. J. Oper. Res. Soc..

[26] Liu H., Ren H., Wu Z., Xu H., Zhang S., Li J., Hou L., Chi R., Zheng H., Chen Y. (2021). CT radiomics facilitates more accurate diagnosis of COVID-19 pneumonia: Compared with CO-RADS. J. Transl. Med..

[27] Shan F., Gao Y., Wang J., Shi W., Shi N., Han M., Xue Z., Shen D., Shi Y. (2020). Abnormal Lung Quantification in Chest CT Images of COVID-19 Patients with Deep Learning and its Application to Severity Prediction. Med. Phys..

[28] Lanza E., Muglia R., Bolengo I., Santonocito O.G., Lisi C., Angelotti G., Morandini P., Savevski V., Politi L.S., Balzarini L. (2020). Quantitative chest CT analysis in COVID-19 to predict the need for oxygenation support and intubation. Eur. Radiol..

[29] Ye Z., Rochwerg B., Wang Y., Adhikari N.K., Murthy S., Lamontagne F., Fowler R.A., Qiu H., Wei L., Sang L. (2020). Treatment of patients with nonsevere and severe coronavirus disease 2019: An evidence-based guideline. Can. Med. Assoc. J..

[30] World Health Organization (2020). Operational Considerations for Case Management of COVID-19 in Health Facility and Community: Interim Guidance, 19 March 2020.

[31] Chinese Medical Association Intensive Medicine Branch, Intensive Medicine Physician Branch of Chinese Medical Doctor Association, Professional Committee of Critical Care Medicine, Chinese Society of Pathophysiology (2020). Recommendations from experts on the management of severe novel coronavirus pneumonia. Chin. J. Crit. Care Med..

[32] Zu Z.Y., Jiang M.D., Xu P.P., Chen W., Ni Q.Q., Lu G.M., Zhang L.J. (2020). Coronavirus Disease 2019 (COVID-19): A Perspective from China. Radiology.

[33] Emanuel E.J., Persad G., Upshur R., Thome B., Parker M., Glickman A., Zhang C., Boyle C., Smith M., Phillips J.P. (2020). Fair Allocation of Scarce Medical Resources in the Time of Covid-19. N. Engl. J. Med..

[34] Mithal A., Jevalikar G., Sharma R., Singh A., Farooqui K.J., Mahendru S., Krishnamurthy A., Dewan A., Budhiraja S. (2021). High prevalence of diabetes and other comorbidities in hospitalized patients with COVID-19 in Delhi, India, and their association with outcomes. Diabetes Metab. Syndr. Clin. Res. Rev..

[35] Pathania A.S., Prathipati P., Abdul B.A., Chava S., Katta S.S., Gupta S.C., Gangula P.R., Pandey M.K., Durden D.L., Byrareddy S.N. (2021). COVID-19 and Cancer Comorbidity: Therapeutic Opportunities and Challenges. Theranostics.

